# A multi-step genomic approach prioritized TBKBP1 gene as relevant for multiple sclerosis susceptibility

**DOI:** 10.1007/s00415-022-11109-8

**Published:** 2022-05-12

**Authors:** Melissa Sorosina, Nadia Barizzone, Ferdinando Clarelli, Santosh Anand, Sara Lupoli, Erika Salvi, Eleonora Mangano, Roberta Bordoni, Tina Roostaei, Elisabetta Mascia, Miriam Zuccalà, Domizia Vecchio, Paola Cavalla, Silvia Santoro, Laura Ferrè, Alen Zollo, PROGEMUS, Lucia Florio, Paolo Ragonese, Alberto Gajofatto, Elio Scarpini, Domenico Caputo, Claudio Gasperini, Franco Granella, Paola Cavalla, Roberto Bergamaschi, Giovanni Ristori, Claudio Solaro, Filippo Martinelli Boneschi, Francesco Passantino, Maura Pugliatti, Antonio Gallo, Laura Brambilla, Marinella Clerico, Fioravante Capone, Maria Trojano, Cristina Barlassina, Daniele Cusi, Vittorio Martinelli, Giancarlo Comi, Maurizio Leone, Massimo Filippi, Nikolaos A. Patsopoulos, Philip L. De Jager, Gianluca De Bellis, Federica Esposito, Sandra D’Alfonso, Filippo Martinelli Boneschi

**Affiliations:** 1 Laboratory of Human Genetics of Neurological Disorders, Division of Neuroscience, IRCCS San Raffaele Scientific Institute, 20132 Milan, Italy; 2 Department of Health Sciences, Interdisciplinary Research Center of Autoimmune Diseases (IRCAD), University of Eastern Piedmont, Avogadro University, 28100 Novara, Italy; 3 Department of Informatics, Systems and Communications (DISCo), University of Milano-Bicocca, Milan, Italy; 4 Department of Health Sciences, University of Milan, 20139 Milan, Italy; 5 Neuroalgology Unit, Fondazione IRCCS Istituto Neurologico “Carlo Besta”, 20133 Milan, Italy; 6 National Research Council of Italy, Institute for Biomedical Technologies, Segrate, 20090 Milan, Italy; 7 Center for Translational and Computational Neuroimmunology, Department of Neurology and the Taub Institute for Research On Alzheimer’s Disease and the Aging Brain, Columbia University Irving Medical Center, New York, NY 10032 USA; 8 MS Centre, SCDU Neurology, AOU Maggiore Della Carità, Department of Translational Medicine, Interdisciplinary Research Center of Autoimmune Diseases (IRCAD), University of Eastern Piedmont Avogadro, 28100 Novara, Italy; 9 MS Center, Department of Neuroscience and Mental Health, City of Health and Science University Hospital of Torino, 10126 Turin, Italy; 10 Neurology Unit, IRCCS San Raffaele Scientific Institute, 20132 Milan, Italy; 11 Department of Pathophysiology and Transplantation (DEPT), Dino Ferrari Centre, Neuroscience Section, University of Milan, 20122 Milan, Italy; 12 Bio4Dreams, Business Nursery for Life Sciences, Piazzale Principessa Clotilde 4/A, 20121 Milan, Italy; 13 SC Neurologia, Dipartimento Di Scienze Mediche, IRCCS Casa Sollievo Della Sofferenza, San Giovanni Rotondo, Italy; 14 Vita-Salute San Raffaele University, 20132 Milan, Italy; 15 Neuroimaging Research Unit, Division of Neuroscience, IRCCS San Raffaele Scientific Institute, 20132 Milan, Italy; 16 Neurophysiology Unit, IRCCS San Raffaele Scientific Institute, IRCCS San Raffaele Scientific Institute, 20132 Milan, Italy; 17 Systems Biology and Computer Science Program, Ann Romney Center for Neurological Diseases, Department of Neurology, Brigham and Women’s Hospital, Boston, MA 02115 USA; 18 Division of Genetics, Department of Medicine, Harvard Medical School, Brigham and Women’s Hospital, Boston, MA 02115 USA; 19 Harvard Medical School, Boston, MA 02115 USA; 20 Broad Institute of Harvard and Massachusetts Institute of Technology, Cambridge, MA USA; 21 Neurology Unit and MS Centre, Foundation IRCCS Ca’ Granda Ospedale Maggiore Policlinico, Via Francesco Sforza 35, 20122 Milan, Italy

**Keywords:** Multiple sclerosis, Genetics, Susceptibility, TBKBP1, Genome-wide association study

## Abstract

**Background:**

Over 200 genetic loci have been associated with multiple sclerosis (MS) explaining ~ 50% of its heritability, suggesting that additional mechanisms may account for the “missing heritability” phenomenon.

**Objective:**

To analyze a large cohort of Italian individuals to identify markers associated with MS with potential functional impact in the disease.

**Methods:**

We studied 2571 MS and 3234 healthy controls (HC) of continental Italian origin. Discovery phase included a genome wide association study (1727 MS, 2258 HC), with SNPs selected according to their association in the Italian cohort only or in a meta-analysis of signals with a cohort of European ancestry (4088 MS, 7144 HC). Top associated loci were then tested in two Italian cohorts through array-based genotyping (903 MS, 884 HC) and pool-based target sequencing (588 MS, 408 HC). Finally, functional prioritization through conditional eQTL and mQTL has been performed.

**Results:**

Top associated signals overlap with already known MS loci on chromosomes 3 and 17. Three SNPs (rs4267364, rs8070463, rs67919208), all involved in the regulation of *TBKBP1*, were prioritized to be functionally relevant.

**Conclusions:**

No evidence of novel signal of association with MS specific for the Italian continental population has been found; nevertheless, two MS loci seems to play a relevant role, raising the interest to further investigations for *TBKBP1* gene.

**Supplementary Information:**

The online version contains supplementary material available at 10.1007/s00415-022-11109-8.

## Introduction

Multiple Sclerosis (MS) is a complex autoimmune disease caused by the interplay of multiple genetic and environmental factors [1]. Large genome wide association studies (GWAS) provided important clues for the identification of genetic causes with more than 233 loci being discovered [2–4]. Although they are robustly associated, the identification of causative variants and the functional mechanism influencing MS pathogenesis has been identified only in few cases [5, 6]. Moreover, these studies rely on the inclusion of different populations to reach very large cohorts necessary to detect small effects like those observed for MS; however, different frequencies of disease variants across the different populations may mask the existence of additional associations. This was clearly evidenced in a GWAS in Sardinians, a genetically isolated population, which led to the identification of a novel MS variant in the *TNFSF13B* gene, whose allelic frequency allows its identification only in Sardinians and Southern European populations [6].

In the present project, we aim to analyse a large cohort of more than 5500 Italian individuals to identify markers associated with MS with potential functional impact in the disease. To boost the power of the analysis, we meta-analysed association results from different cohorts and leveraged quantitative molecular traits on adequately large resources for functional SNPs prioritization.

## Materials and methods

### Subjects

A total of 2571 Italian MS patients and 3234 unrelated HC have been recruited across Italian centres by three consortia (PROGRESSO, PROGEMUS and HYPERGENES). All participants were of Caucasian ancestry.

Based on the genotyping platform used for the genotyping, this entire set of samples was divided in four cohorts:

- ITA_GWAS_: it was composed of 776 MS and 1315 HC. MS samples were previously genotyped in the context of the International Multiple Sclerosis Consortium (IMSGC) at genome-wide level at the Wellcome Trust Sanger Institute using the Illumina^®^ Human660-Quad chip [2]. Healthy individuals (mean (± SD) age of 53.4 ± 9.9 years and no abnormal findings on physical and neurological examination) have been collected within the HYPERGENES project (European Network for Genetic-Epidemiological Studies; www.hypergenes.eu) [7] in Italy and genotyped at the University of Milan, using the Illumina^®^ Infinium 1 M-duo BeadChips.
- ITA_iChip_: it was composed of 964 MS patients and 1054 matched controls non-overlapping with the ITA_GWAS_ cohort. Samples were previously genotyped in the context of the IMSGC at the Wellcome Trust Sanger Institute using the Illumina^®^ Immunochip covering 196,524 SNPs and 184 non-HLA genomic regions with potential immunological function [3].
- ITA_OA_: it was composed of 917 MS patients and 924 controls. Samples were genotyping in the present study for selected SNPs using custom and pre-designed TaqMan assays (Thermo Fisher Scientific, Waltham, USA) assembled on two OpenArray chips run on the TaqMan Open Array Genotyping System (Thermo Fisher Scientific, Waltham, USA) or, in the context of the IMSGC, with the Illumina MS replication chip (Illumina, San Diego, USA), a custom array covering more than 300,000 SNPs localized in MS associated loci [4]
- ITA_NGS_: subjects were chosen starting from the ITA_GWAS_ and ITA_iChip_ cohorts based on their genetic risk burden [8], estimated as a weighted sum of risk alleles in non-HLA known MS-associated loci [9–11]; 600 MS patients with a higher genetic risk (based on the distribution of the entire Italian cohort) were selected together with age- and sex-matched 408 HC, according to sex and Principal Components (PCs) to account for population stratification. ITA_NGS_ cohort underwent pooled-sequencing as previously described [12] using the Agilent SureSelect target enrichment method (Agilent Technologies, Santa Clara, USA) according to the manufacturer’s protocol.

The four cohorts are partially overlapping, as shown in Online Resource 1 fig. S1.

Demographic information for the mentioned cohorts is listed in Table 1, while details on genetic data generation are described in the Online Resource 1.

**Table 1:** Baseline demographic and clinical features of Italian individuals included in the study

	ITA_GWAS_		ITA_iChip_		ITA_OA_		ITA_NGS_	
	MS	HC	MS	HC	MS	HC	MS	HC
*n*	776	1315	964	1054	917	924	600	408
Sex								
Males	277	878	323	610	294	591	238	172
Females	499	437	641	444	623	333	362	236
Disease course								
BOMS	561	–	875	–	709	–	499	–
PPMS	125	–	58	–	86	–	61	–
CIS	7	–	9	–	5	–	6	–
Undefined	83	–	22	–	117	–	34	–
Age at onset (average ± SD)	31.7 ± 10.1	–	30.3 ± 10.0	–	31.0 ± 10.3	–	30.2 ± 10.0	–
Disease duration (average ± SD), years	11.3 ± 8.5	–	11.2 ± 7.5	–	11.0 ± 8.7	–	11.3 ± 8.2	–

*BOMS* Bout-onset MS patients (including relapsing–remitting and secondary-progressive), *PPMS* progressive MS patients, *CIS* Clinically isolated syndrome, *undefined* MS patients with no definition of diagnosis

Additionally to the Italian cohorts, a total of 4088 MS patients and 7,144 HC of European descent (“NonIT-EUR” cohort) have been included in the analyses for the “meta-analysis approach” (see below). This represents a combination of 6 individual sample collections previously described [13].

All patients were diagnosed according to Poser and McDonald criteria and further revisions [14].

### Design of the study

**Fig. 1:**
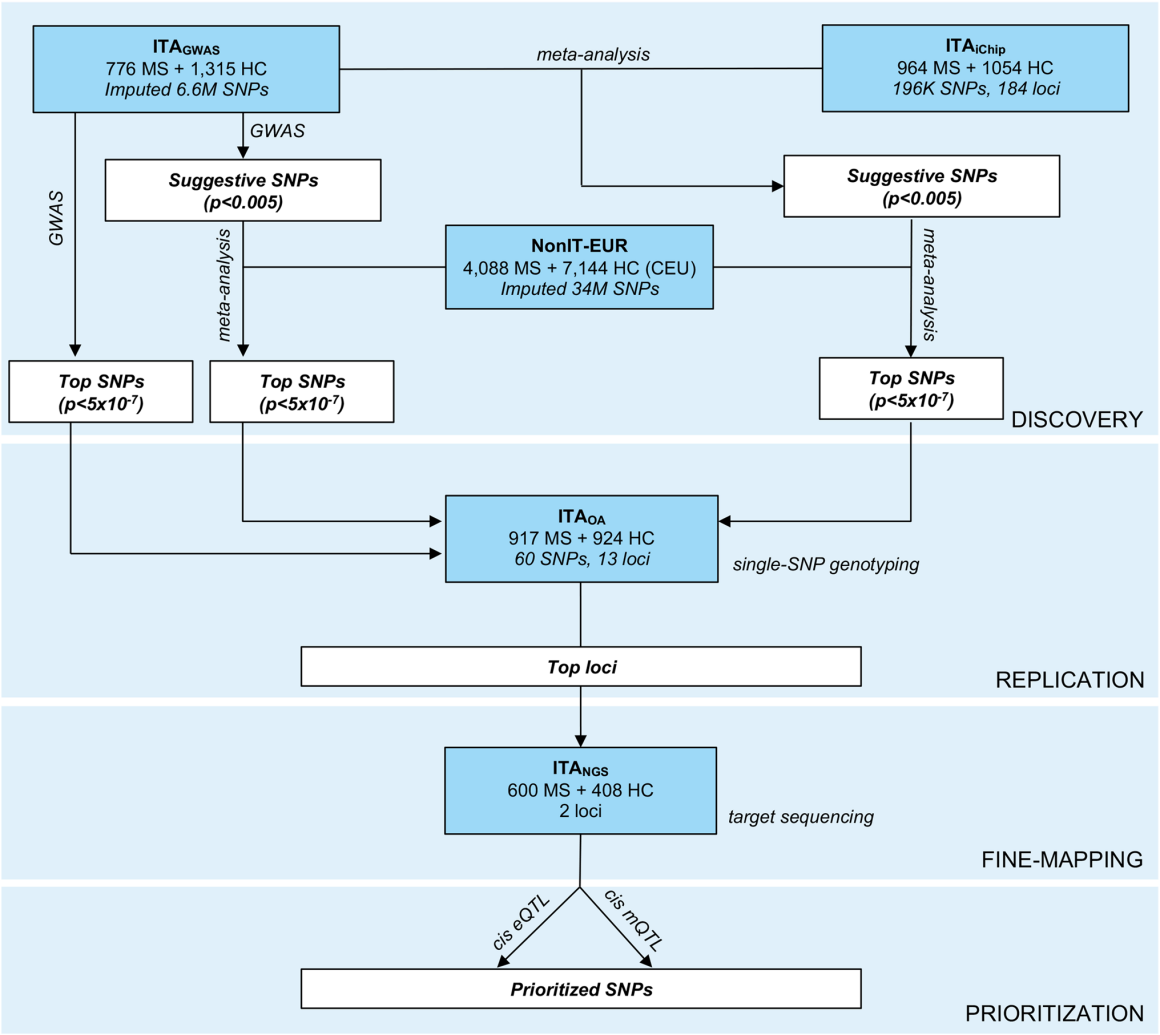
Workflow of the study. The overall workflow of the study is shown. The discovery phase includes two Italian cohorts (ITA_GWAS_ and ITA_iChip_) from which a set of top MS associated SNPs were identified through a genome-wide approach (in the ITA_GWAS_ cohort only) or a meta-analytic approach including also the NonIT-EUR dataset. The identified top SNPs (*P* < 5 × 10^– 7^) were further tested for replication in a third independent Italian cohort (ITA_OA_) leading to the confirmation of two associated loci. Those loci were sequenced (ITA_NGS_ cohort) and the associated SNPs underwent prioritization through conditional *cis* eQTL and mQTL analyses

Figure 1 shows the workflow of the study. Briefly, we followed a multi-step approach including a discovery step at genome-wide level followed by replication and fine-mapping through next-generation sequencing of the top-associated loci. Finally, the effect of the identified variants on gene expression and DNA methylation was evaluated to prioritize markers with potential functional role.

#### Discovery

We initially investigated the presence of association signals specific for the Italian population by performing a GWAS in the ITA_GWAS_ cohort alone (here called the “genome-wide Italian approach”), this led to the selection of SNPs (*P* < 5 × 10^– 7^) to further replicate in the replication step.

An alternative approach (here called the “meta-analysis approach”) was designed to increase the sample size of the discovery phase and detect association signals with a lower impact on MS susceptibility not detectable when studying only the ITA_GWAS_ cohort. Specifically, two additional cohorts were included in the study design: the ITA_iChip_, composed by Italian individuals genotyped at relevant immunological genomic regions, and the NonIT-EUR dataset, which represents a large cohort of non-Italian individuals for which genome-wide genotyping data are available. According to this approach, we selected SNPs with suggestive evidence of association (*P* < 0.005) in the ITA_GWAS_ cohort alone (to detect signals at genome-wide level), as well as performing a meta-analysis between ITA_GWAS_ and ITA_iChip_ cohorts. A meta-analysis with the NonIT-EUR dataset was then performed for the selected signals and significant SNPs (*P* < 5 × 10^– 7^) were considered for replication.

#### Replication

We defined loci for replication as described in the Online Resource 1. Due to the known strong associations within the MHC region with MS [4], SNPs located in the HLA locus were excluded.

Genotyping was performed using TaqMan assays and the TaqMan Open Array Genotyping System (Thermo Fisher Scientific, Waltham, USA) or the MS Chip [4] (Illumina, San Diego, USA). Single-SNP logistic regression using sex as covariate was conducted under additive model of inheritance. Meta-analyses between the discovery and replication cohorts were performed assuming a fixed-effect model.

#### Fine mapping with next generation sequencing (NGS)

We sequenced the Linkage Disequilibrium (LD) blocks containing the replicated associated signals in the ITA_NGS_ cohort using the Agilent SureSelect kit (Agilent Technologies, Santa Clara, USA) and a pool-based approach on the GaIIx platform (Illumina, San Diego, USA). Bioinformatics pipeline and variant calling were performed as described [12]. Fisher exact test comparing MS and HC was performed on allelic counts, no correction based on sex or ancestry was included since HC were individually matched with MS patients, as described in the “Subjects” paragraph. For methodological details see Online Resource 1.

#### Prioritization

Identified associated variants were prioritized according to their *cis* effect on expression or CpG methylation by performing conditional *cis*-eQTL and conditional *cis*-mQTL analyses. C*is*-eQTL analysis was performed taking advantage of the GTEx Portal v6p [15] dataset (release phs000424.v6.p1) for 7 MS-related tissues (whole blood, Brain Frontal_Cortex (BA9), Brain Cortex, Cerebellar Hemisphere, Cerebellum, Anterior cingulate cortex (BA24), Hypothalamus). *Cis*-mQTL analysis was performed on CD4^+^ T-cells collected from 156 MS patients within the Comprehensive Longitudinal Investigation of Multiple Sclerosis at the Brigham and Women's Hospital (CLIMB study [16, 17]). Both analyses were performed using QTLtools [18]. For details see Online Resource 1.

## Results

### Discovery

As described in the method section, the “genome-wide Italian approach” includes the ITA_GWAS_ cohort as discovery dataset. After quality control (QC), the association analysis was performed in a final dataset of 737 MS and 1291 HC individuals and ~ 6.6 million SNPs. Seventy-one variants reached a *p* value of association < 5 × 10^–7^, within 26 distinct loci (Online Resource 2, Online Resource 1 Fig. S2).

The “meta-analysis approach” includes both the ITA_GWAS_ and ITA_iChip_ cohorts as discovery datasets, with signals with suggestive evidence of association (*P* < 0.005) selected to be meta-analysed with the NonIT-EUR. Regarding the ITA_GWAS_ cohort alone, 32,309 SNPs (*P* < 0.005) were meta-analysed with the NonIT-EUR leading to a list of 49 SNPs with a p-value of association < 5 × 10^– 7^ (Online Resource 3A). The meta-analysis of the two Italian discovery datasets led to the identification of 1373 SNPs suggestive of association (*P* < 0.005). Meta-analysis of these SNPs with the NonIT-EUR dataset resulted in 21 SNPs with a *p* value < 5 × 10^– 7^ (Online Resource 3B) within 5 loci. Overall, and given the fact that some signals overlapped between the different analyses, 127 signals across 35 loci were selected (Online Resource 2 and Online Resource 3).

### Replication

Starting from the 127 signals identified through the discovery analyses (Online Resource 2 and Online Resource 3), we selected 60 SNPs for replication as representative of the identified genomic loci (see Online Resource 1 for details and Online Resource 2 and Online Resource 3 for the final list). After genotyping and QC (see Online Resource 1 for details), 48 SNPs within 30 loci were tested for association in the ITA_OA_ dataset (Table 2).

**Table 2:** Association results in the replication sample

			Replication				Meta-analysis discovery and replication				
SNP	LOCUS	A1	OR	L95	U95	*P*	OR	L95	U95	*P*	*I*^2^
Table 2A											
rs1595367	locus 27	T	0.80	0.66	0.98	0.03	1.26	1.11	1.44	5.12E-04	97.04
rs1914544	locus 17	C	0.87	0.75	1.02	0.08	1.20	1.08	1.33	7.40E-04	96.94
rs13151412	locus 12	T	0.88	0.75	1.02	0.09	1.15	1.03	1.28	1.29E-02	95.88
rs11492289	locus 18	G	0.89	0.78	1.02	0.10	0.82	0.74	0.91	1.35E-04	97.89
rs6993986	locus 15	C	0.90	0.77	1.04	0.15	0.84	0.75	0.93	1.17E-03	96.58
rs12872924	locus 23	T	0.90	0.78	1.04	0.17	0.85	0.76	0.95	2.75E-03	96.01
rs61500084	locus 11	A	0.91	0.78	1.05	0.19	1.16	1.05	1.28	4.67E-03	95.06
rs35493137	locus 26	T	1.15	0.92	1.44	0.21	1.51	1.31	1.74	1.34E-08	89.3
rs12873058	locus 23	T	0.91	0.78	1.06	0.22	1.18	1.06	1.32	1.87E-03	95.79
rs6011709	locus 31	T	1.11	0.94	1.31	0.22	1.37	1.22	1.53	6.81E-08	91.15
rs12453667	locus 26	G	0.74	0.45	1.21	0.23	1.55	1.33	1.80	1.22E-08	89.61
rs9852162	locus 11	T	0.92	0.79	1.06	0.24	1.20	1.08	1.32	3.77E-04	95.99
rs7316186	locus 22	A	0.93	0.81	1.06	0.28	0.80	0.73	0.89	1.39E-05	97.45
rs3589	locus 31	T	1.09	0.93	1.29	0.28	1.30	1.15	1.47	1.61E-05	89.49
rs2278819	locus 14	G	0.88	0.69	1.11	0.29	0.77	0.65	0.90	9.84E-04	95.01
rs663743	locus 5	G	1.07	0.92	1.24	0.39	0.78	0.70	0.87	5.21E-06	91.18
rs2510066	locus 5	C	1.07	0.92	1.24	0.39	0.78	0.70	0.87	4.72E-06	91.23
rs2508634	locus 21	T	0.92	0.73	1.14	0.44	1.27	1.07	1.50	5.60E-03	94.6
rs6594535	locus 3	T	1.05	0.91	1.22	0.48	1.24	1.12	1.38	3.53E-05	89.31
rs11229374	locus 19	T	0.93	0.76	1.14	0.49	0.79	0.69	0.91	9.04E-04	94.06
rs2933291	locus 10	G	1.06	0.89	1.27	0.50	1.30	1.14	1.49	7.62E-05	90.33
rs528650	locus 16	A	1.18	0.68	2.07	0.56	0.46	0.33	0.64	5.94E-06	86.37
rs12972735	locus 28	T	0.96	0.83	1.11	0.57	1.20	1.09	1.32	2.58E-04	94.17
rs548827	locus 16	T	1.15	0.66	2.02	0.63	0.46	0.33	0.65	7.59E-06	87
rs76053860	locus 24	G	0.97	0.65	1.45	0.88	0.55	0.41	0.72	1.84E-05	94.2
rs6875879	locus 13	A	0.99	0.85	1.16	0.93	1.23	1.10	1.36	1.65E-04	92.37
16:89463366	locus 25	R	0.99	0.86	1.15	0.93	1.21	1.09	1.34	3.64E-04	92.64
rs9927904	locus 25	G	1.00	0.87	1.16	0.96	1.21	1.09	1.34	3.01E-04	91.94
rs6591385	locus 20	T	0.00	0.00	Inf	1.00	2.06	1.57	2.70	1.70E-07	0

			Replication				Meta-analysis discovery and replication					Meta-analysis with only Italians cohorts				
SNP	LOCUS	A1	OR	L95	U95	*P*	OR	L95	U95	*P*	*I*^2^	OR	L95	U95	*P*	*I*^2^
Table 2B																
**rs338603**	**locus 1**	**C**	**1.27**	**1.11**	**1.46**	**8.01E-04**	**1.21**	**1.14**	**1.28**	**1.77E-10**	**0**	**1.29**	**1.17**	**1.42**	**3.73E-07**	**0**
rs8070463	locus 8	T	1.24	1.08	1.44	3.43E-03	1.21	1.14	1.28	1.79E-11	0	1.25	1.13	1.38	1.72E-05	0
rs438613	locus 1	C	1.23	1.06	1.42	5.74E-03	1.20	1.13	1.27	9.68E-10	0	1.27	1.15	1.40	2.67E-06	0
rs741174	locus 7	C	1.22	1.06	1.41	7.19E-03	1.18	1.11	1.24	5.48E-09	0	1.22	1.11	1.35	7.59E-05	0
rs741175	locus 7	T	1.19	1.03	1.37	1.52E-02	1.16	1.10	1.22	9.70E-09	0	1.20	1.09	1.33	1.80E-04	0
rs4267364	locus 8	A	1.19	1.02	1.39	2.44E-02	1.18	1.11	1.24	1.07E-08	0	1.22	1.10	1.36	2.37E-04	0
rs1883832	locus 9	T	1.16	1.00	1.36	0.051	1.17	1.11	1.24	4.02E-08	0	1.20	1.08	1.34	8.29E-04	0
rs6065926	locus 9	A	1.16	0.99	1.35	0.059	1.17	1.11	1.24	3.18E-08	0	1.21	1.09	1.35	4.67E-04	0
rs2729590	locus 6	T	1.20	0.98	1.48	0.080	1.37	1.23	1.53	1.16E-08	52.8	1.41	1.22	1.62	2.56E-06	75.9
rs56038902	locus 33	T	1.08	0.92	1.27	0.346	1.19	1.11	1.27	3.61E-07	43.9	1.18	1.05	1.32	4.28E-03	60.5
rs55857387	locus 33	T	1.07	0.91	1.26	0.404	1.19	1.11	1.27	2.43E-07	53.6	1.19	1.06	1.33	2.86E-03	71.4
rs529279	locus 3	C	1.03	0.87	1.22	0.722	1.14	1.08	1.20	1.32E-06	55.2	1.19	1.08	1.32	6.59E-04	86.1
rs2729589	locus 6	T	1.12	0.58	2.16	0.726	1.33	1.20	1.48	6.37E-08	0	1.45	1.21	1.73	4.46E-05	0

			Replication				Meta-analysis discovery and replication					Meta-analysis with only Italians cohorts				
SNP	LOCUS	A1	OR	L95	U95	*P*	OR	L95	U95	*P*	*I*^2^	OR	L95	U95	*P*	*I*^2^
Table 2c																
**rs669607**	**locus 1**	**C**	**1.23**	**1.07**	**1.42**	**4.47 × 10**^**– 3**^	**1.18**	**1.12**	**1.24**	**1.31 × 10**^**– 10**^	**0**	**1.23**	**1.13**	**1.33**	**6.01 × 10**^**– 7**^	**0**
rs11878602	locus 34	C	1.23	1.03	1.46	2.04 × 10^– 2^	1.16	1.10	1.23	3.18 × 10^– 8^	0	1.20	1.10	1.32	9.54 × 10^– 5^	25.8
rs1870071	locus 34	C	1.22	1.03	1.46	2.24 × 10^– 2^	1.16	1.10	1.23	3.04 × 10^– 8^	0	1.21	1.10	1.33	8.58 × 10^– 5^	32.0
rs1883832	locus 9	T	1.17	1.00	1.36	4.62 × 10^– 2^	1.16	1.10	1.22	1.40 × 10^– 8^	0	1.17	1.08	1.27	2.82 × 10^– 4^	0
rs6065926	locus 9	A	1.16	1.00	1.36	0.053	1.16	1.11	1.23	8.71 × 10^– 9^	0	1.18	1.08	1.28	1.32 × 10^– 4^	0
rs11079784	locus 8	C	1.11	0.96	1.29	0.150	1.16	1.11	1.22	8.21 × 10^– 10^	0	1.15	1.07	1.25	4.66 × 10^– 4^	0
rs11870935	locus 8	A	1.10	0.95	1.27	0.200	1.15	1.10	1.21	1.44 × 10^– 8^	0	1.14	1.06	1.24	1.07 × 10^– 3^	0
rs11256593	locus 35	T	1.06	0.92	1.23	0.409	1.15	1.10	1.20	3.94 × 10^– 9^	11.3	1.12	1.04	1.22	4.61 × 10^– 3^	0

For each SNP, the association data in the replication cohort and results from the meta-analyses between discovery and replication cohorts (including and not including the NonIT-EUR cohort) are reported. Results for the genome-wide Italian approach are reported in the upper panel (A), while results for the approaches including the NonIT-EUR are reported in panel B (discovery sample derived from ITA_GWAS_ alone) and C (SNPs identified through the meta-analysis between ITA_GWAS_ and ITA_iChip_)

*A1*: reference allele (the same as defined in Table 1); *OR* : odds ratio; *L95*: lower-bound of 95%-confidence interval; U95 upper bound of 95%-confidence interval; P: *P* value of association; *I*^2^ heterogeneity index.

The SNPs with an association beyond the multiple testing correction (Bonferroni threshold of *P* value: 1.7 × 10^– 3^, 3.6 × 10^– 3^, 6.25 × 10^– 3^ respectively for A, B and C) are highlighted in bold. No significant association was found in the replication cohort for SNPs selected through the Italian genome-wide approach

No SNPs identified through the “genome-wide Italian approach” showed evidence of association, while according to the “meta-analysis approach” (thus starting from the two ITA_GWAS_ and ITA_iChip_ cohorts), the locus 1 on chromosome 3 and locus 8 on chromosome 17 confirmed their association, with rs338603 (OR = 1.27, *P* = 8.01 × 10^− 4^) and rs669607 (OR = 1.23, *P* = 4.47 × 10^− 3^) and rs8070463 (OR = 1.24, *P* = 3.43 × 10^− 3^) as the top associated SNPs. These associations were confirmed also performing a meta-analysis excluding the NonIT-EUR cohort and including only dataset of Italian origin (*P* = 3.73 × 10^– 7^, 6.01 × 10^– 7^ and 1.72 × 10^− 5^ respectively for rs338603, rs669607 and rs8070463) (Table 2, Online Resource 1 Fig. S3).

### Fine mapping

**Fig. 2:**
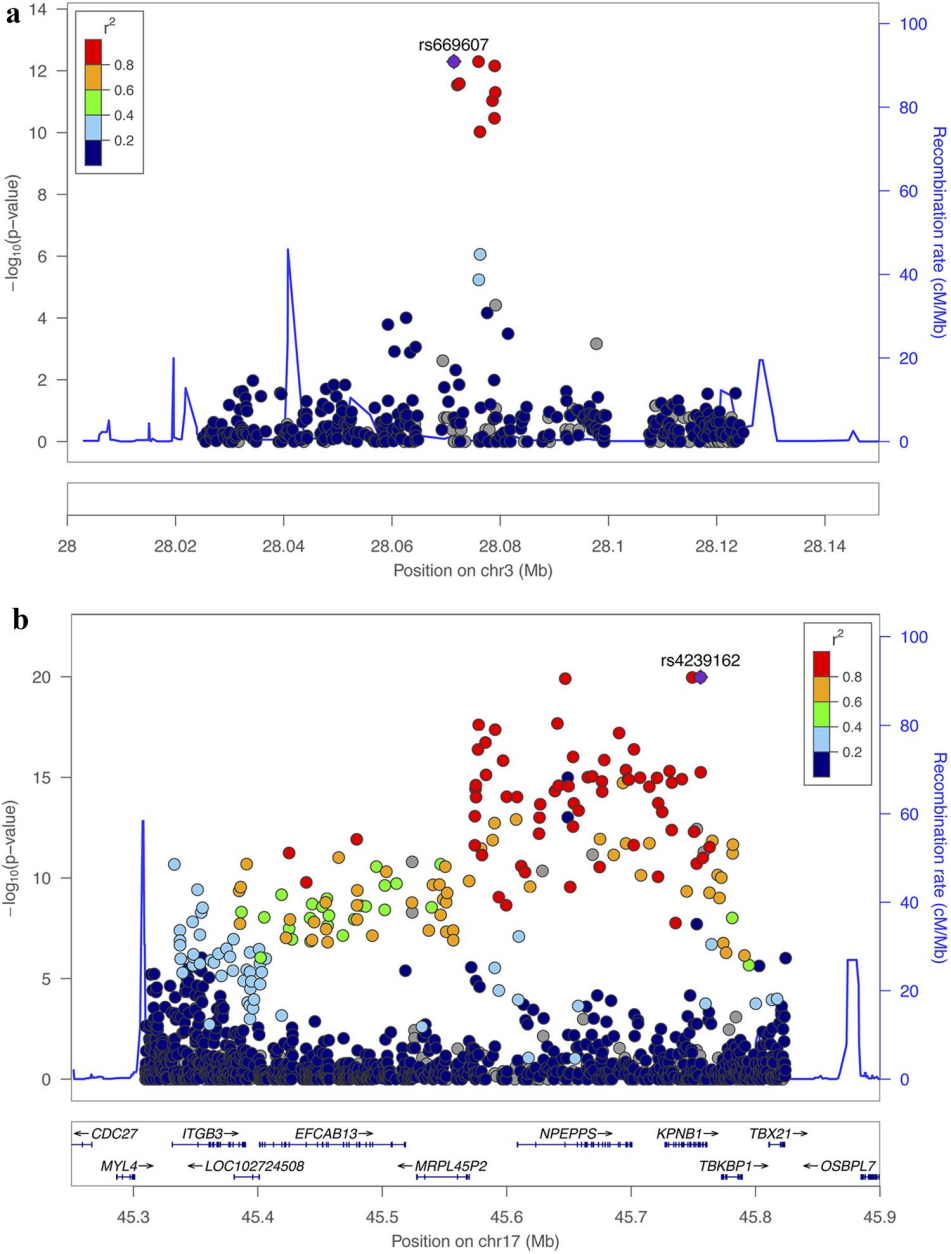
Regional association plots of replicated loci in ITA_NGS_ cohort. Regional association plots for locus 1 on chromosome 3 (**a**) and locus 8 on chromosome 17 (**b**) designed with LocusZoom (http://locuszoom.sph.umich.edu) are shown. For each locus, the negative log-transformed *P* values (left y-axis) and the recombination rate (right y-axis) according to the 1000 Genomes Project (Nov 2014, Europeans only) are plotted over the genomic position (hg19). Each symbol represents one SNP, with the most associated SNP marked in purple and shading of the other points based on the linkage disequilibrium with the top SNP. Positions of genes, if present, are shown below the plot

The replicated locus on chromosome 3 spans from position 28024996 to 28124996 (hg19 assembly). This is an intergenic region including the non-coding genes *LINC01980* (~ 123 kb) and LINC01981 (~ 150 kb) and the coding genes *EOMES* (~ 160 Kb) and *CMC1* (~ 150 kb) as the closest downstream genes. A total of 608 variants were identified through the sequencing approach. Comparing to the post-QC imputed ITA_GWAS_ dataset, the inclusion of a sequencing step allowed to investigate additional 74 common variants (MAF ≥ 0.01), as well as 636 rare variants (Minor allele Frequency (MAF) < 0.01), while 109 were excluded and not tested in the ITA_NGS_ cohort due to the stringent QC applied. Fisher’s exact test on sequenced data revealed the association of 12 variants (Bonferroni-adjustment alpha = 5.87 × 10^– 5^). The most associated SNPs were rs669607 and rs427221 (*P* = 5.02 × 10^− 13^) (Fig. 2a, Online Resource 4).

The replicated locus on chromosome 17 spans a large LD block of ~ 500 kb (chr17:45309693-45824486, hg19 assembly) which includes 8 genes: *ITGB3*, *EFCAB13* (*C17orf57*), *NPEPPS*, *KPNB1*, *TBKBP1*, *TBX21*, *MRPL45P2* (pseudogene) and *LOC102724508* (a non-coding RNA, with alias *THCAT158*). In this region, a total of 2,428 variants were identified through the sequencing approach. Comparing to the post-QC imputed ITA_GWAS_ dataset, the inclusion of the fine mapping step allowed to investigate additional 446 common variants and 2,395 rare variants, while 341 SNPs were not tested in the ITA_NGS_ cohort for the sequencing QC. Applying a pipeline which allows an accurate allele frequency estimation from NGS pooled data [12] to perform the association analysis, we found that 202 SNPs were significantly associated after Bonferroni-adjustment (alpha = 1.57 × 10^− 5^) (Fig. 2b, Online Resource 4). The strongest association signal was rs4239162 (*P* = 1.04 × 10^− 20^), intronic to *KPNB1*.

### Locus on chromosome 3

The locus on chromosome 3 is a small intergenic genomic region, with apparently no evidence of chromatin annotation (Online Resource 1 Fig. S4), except for the presence of sporadic weak enhancers. *Cis* eQTL analysis did not reveal any association between the 12 SNPs identified in the genetic analyses and the expression of nearby genes.

### Locus on chromosome 17: functional SNPs prioritization

**Fig. 3:**
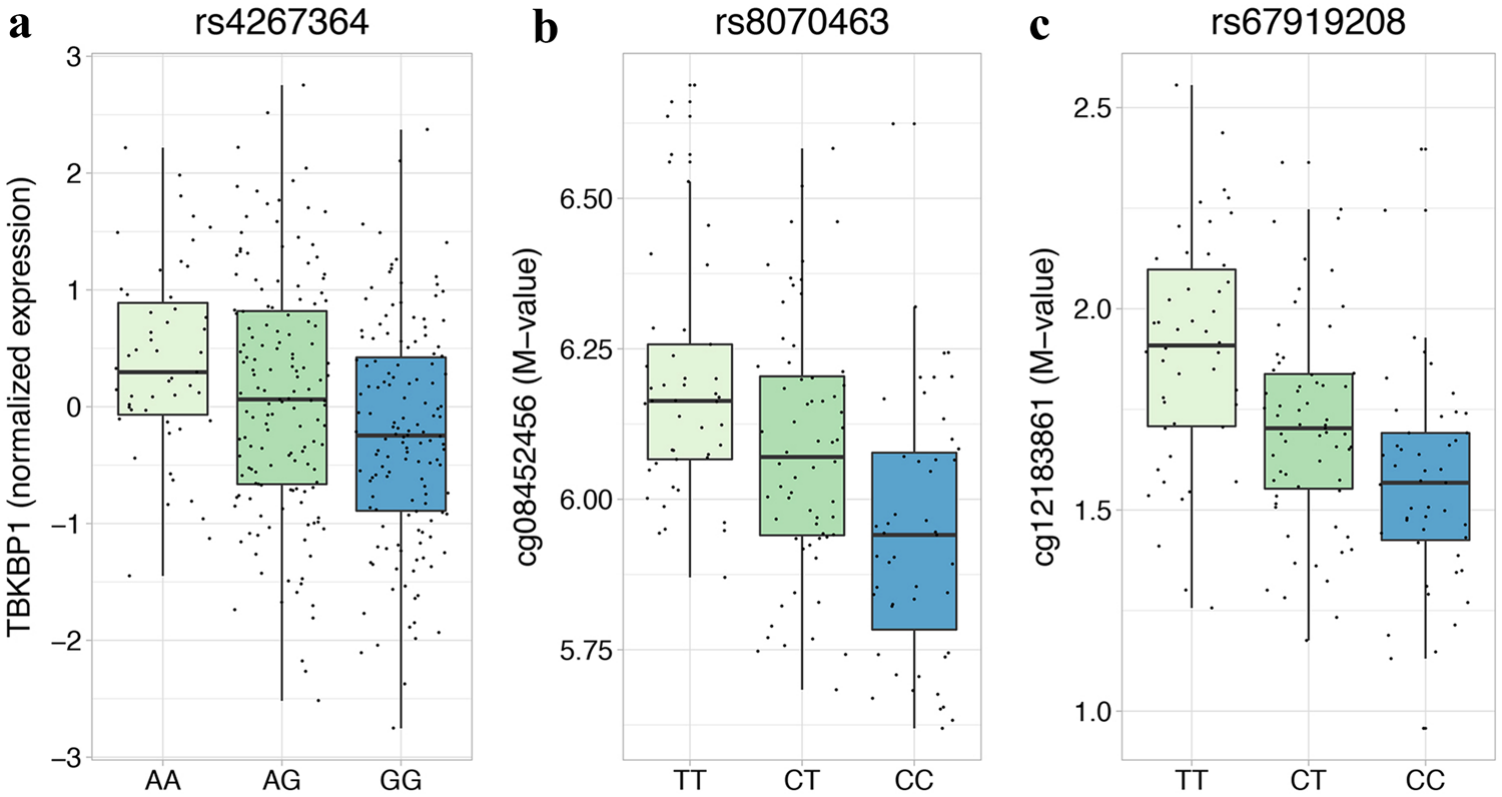
Prioritized eQTL and mQTL effects. eQTL and mQTL effects are depicted in the figures. **a** TBKBP1 normalized expression values in whole blood are plotted against the genotype of rs4267364; **b** cg08452456 *M* values are plotted against the genotype of rs8070463; **c** cg12183861 *M* values are plotted against the genotype of rs67919208. Representation is in box-plot format (Tukey method) with each dot representing one individual

We sought to characterize the identified MS risk variants prioritizing those with a putative functional impact. We then performed a conditional *cis*-eQTL analysis in 7 tissues relevant for MS according to data generated within the GTEx project [15]. As shown in Online Resource 5A, we found 7 SNP-gene pairs associated in the tested tissues. Among them, rs4267364 showed a consistent association with MS in all the steps of the study (Table 3). This SNP is intronic to *TBKBP1* and the MS associated allele (rs4267364_A_) is associated to a higher expression of this gene in blood (Fig. 3a). According to the chromatin state prediction in 14 epigenomes of immune-related cells profiled by the Roadmap Epigenomics [19] and ENCODE [20] projects, this SNP is localized in a putative enhancer, supporting its regulatory function (Online Resource 1 Fig. S5).

**Table 3:** MS associated signals showing independent eQTL and mQTL associations

					eQTL		ITA_GWAS_		Meta-analysis between ITA_GWAS_ and NonIT-EUR		Replication		ITA_NGS_	
SNP	GENE	SNP POSITION	A1	A2	P	SLOPE	P	OR	P	OR	P	OR	P	OR
Table 3A														
rs4267364	TBKBP1	17:45781264	G	A	1.71 × 10^– 23^	− 0.29	3.12 × 10^– 3^	0.81	1.42 × 10^– 7^	0.85	2.44 × 10^– 2^	0.84	9.61 × 10^– 9^	0.58

						mQTL		ITA_GWAS_		Meta-analysis between ITA_GWAS_ and NonIT-EUR		Replication		ITA_NGS_	
SNP	CpG	SNP POSITION	A1	A2	CpG POSITION	P	SLOPE	P	OR	P	OR	P	OR	P	OR
Table 3B															
rs67919208	cg12183861	17:45771873	C	T	17:45773468	1.62 × 10^– 12^	− 0.68	1.07 × 10^– 3^	0.80	1.36 × 10^– 9^	0.84	^a^	–	^b^	–
rs8070463	cg08452456	17:45768836	C	T	17:45786439	2.05 × 10^– 17^	− 0.79	1.54 × 10^– 3^	0.80	1.34 × 10^– 9^	0.84	3.43 × 10^– 3^	0.81	^b^	–

Signals associated with MS according to at least two of the steps performed (discovery: meta-analysis between ITA_GWAS_ and NonIT-EUR *P* < 5 × 10^– 7^; replication: *P* < 3.6 × 10^– 3^, target sequencing: *P* < 1.57 × 10^– 5^) that show also independent eQTL (A) or mQTL (B) association are shown. For the SNP with eQTL effect, the SNP-associated gene pair is indicated, together, in the eQTL panel, with the backward nominal *P* value of association and the backward regression slope referred to A1 in whole blood. For the SNPs with an mQTL association, the SNP-CpG pairs are indicated together, in the mQTL panel, with the backward nominal *P* value of association and the backward regression slope referred to A1. When available, genetic association results are reported. No SNP fulfilling the mentioned criteria is present in the ITA_iChip_ dataset, thus *P* values are not reported

*A1* reference allele for QTL analyses and ORs, *A2* alternative allele, *P* *P* value of association, *OR* Odds ratio

^a^SNP not selected for the replication phase

^b^SNP failing the QC in the sequencing step

To explore additional mechanisms of regulation, we performed a conditional *cis*-mQTL in CD4^+^ cells collected from MS patients. The Online Resource 5B lists the significant SNP-CpG pairs resulted from this analysis. Among the identified mQTL associations, one SNP associated to MS according to the discovery strategy and at least one of the replication steps, rs8070463, was found to be associated also with the methylation status of cg08452456 (Table 3, Fig. 3b), exonic to *TBKBP1*. An additional SNP, rs67919208, associated with the methylation status of cg12183861 (Table 3, Fig. 3c), localized in the 5’UTR region of *TBKBP1,* was also found to be associated with MS (discovery step). Both rs8070463 and rs67919208 had the MS risk variant associated with a higher CpG methylation (Table 3).

All the three prioritized variants suggest an involvement of *TBKBP1* gene, thus we then evaluated the expression of *TBKBP1* across immune cells according to the DICE dataset [21]. As shown in Online Resource 1 fig. S6, TBKBP1 is expressed across several immune cell types, with the highest levels in activated T cells (CD4^+^ and CD8^+^) and Natural Killer cells.

## Discussion

Large scale international studies showed a highly polygenic architecture for MS genetic susceptibility [4]; nonetheless, the identified variants explain ~ 50% of the genetic predisposition to MS, suggesting that additional signals and disease mechanisms exist. This “missing heritability” could be explained, at least partially, by the presence of population-specific associations that cannot be fully captured by array-based international studies, as already shown studying the Sardinian [6] and German populations [22].

To investigate the presence of strong association signals enriched in the Italian population, we initially performed a genome-wide association study (referred along the paper as the “genome-wide Italian approach”) in a cohort of Italian individuals that led us to the identification of several loci potentially associated with MS, most of them never reported in previous studies (Online Resource 2). However, replication did not confirm any of these associations, supporting the idea that no strong signals exist in the continental Italian population. Nevertheless, we can’t exclude that other events occurred, including population stratification, genetic heterogeneity, or an insufficient statistical power [23].

We then followed an alternative approach, exploring also signals with suggestive evidence of association and meta-analysing them with a cohort of European descent with larger adequate sample size and statistical power. The genome-wide Italian approach would have the potential to identify signals with a strong association with MS specific for the Italian population, while the second approach combined the advantage of using an ancestrally homogeneous cohort as the driving cohort of loci selection with the statistical power gained using a larger cohort. Specifically, this second approach, referred as “meta-analysis approach”, highlighted the presence of two loci representing the main results of this effort. Both lie in loci already known to be associated with MS [2–4]: an intergenic genomic region on chromosome 3 (~ 100 Kb) and a large locus of ~ 500 kb on chromosome 17.

To better characterize the two selected loci and to prioritize putative causal variants, we then performed a deep target sequencing followed by functional prioritization, allowing to investigate additional associated SNPs, not covered by arrays or imputation. In the case of the locus on chromosome 3, this analysis led to the identification of 12 associated SNPs, with rs669607 and rs427221 as the top associated ones [2]. No clear function is suggested, since this is an intergenic region with no annotation suggestive of specific regulatory function [4]. Nevertheless, very recently, a regulatory effect on the upstream gene *EOMES* in T cells has been highlighted [24], in line with previous studies supporting a role of this gene in MS [25].

On the contrary, the locus on chromosome 17 is more complex, with several genes and potentially regulatory regions. Sequencing of this region led to the identification of 202 signals of associations with the top associated signals localized in the downstream part of the region, as suggested also by the discovery analysis. This high number of associated signals could be explained by the strong linkage disequilibrium characterizing this locus (Fig. 2b), which further complicates the identification of the causative signal of association. We therefore resorted to exploit regulatory data for prioritization of the identified SNPs. Several studies reported an enrichment of regulatory regions within MS and other complex diseases GWAS loci [24, 26–30], thus we performed a conditional *cis* eQTL analysis to either overcome the presence of the large LD block and to combine the results with the obtained genetic association. This analysis highlighted rs4267364, which is an intronic polymorphism to *TBKBP1*, localized in a putative enhancer. This SNP is associated with the expression of *TBKBP1* in blood, but not in brain-related tissues, with the MS risk allele rs4267364_A_ associated with a higher expression of *TBKBP1* (*P* = 1.71 × 10^– 23^). We also investigated additional mechanisms of regulation performing a conditional *cis* mQTL analysis to identify SNPs associated with the methylation state of CpG in CD4^+^ lymphocytes of MS patients. Several associations were found (Online Resource 5B), with two SNPs, rs8070463 and rs67919208, which were also selected by the genetic approach. These two SNPs were found to be associated with the methylation status of respectively cg08452456, exonic to *TBKBP1*, and cg12183861, localized in the 5’UTR of *TBKBP1*. Interestingly enough, rs8070463 SNP has been previously associated with ankylosing spondylitis, a rare autoimmune condition [31]. It is well established that methylation of regulatory regions is an important mechanism [32]: DNA methylation in promoter regions is known to induce gene silencing [33], while it has been reported that DNA methylation in transcribed regions seems to correlate with an induced gene expression [34]. Given their genomic position, we could hypothesize that the influence on the methylation status of the mentioned CpGs could in turn lead to an upregulation of *TBKBP1*, as suggested by eQTL analysis on GTEx dataset, with the MS risk allele being associated with an upregulation of *TBKBP1* in whole blood (*P* = 1.03 × 10^– 20^ and 2.17 × 10^– 20^ respectively for rs8070463 and rs67919208), although we could not exclude that this reflects a LD with the eQTL leading SNP rs4267364 (*r*^2^ = 0.66 for both the SNPs). Functional experiments testing methylation and expression profiles in same samples are necessary to clarify the direction of the signal.

All of the three prioritized SNPs seems to be involved in the regulation of the expression of *TBKBP1*, suggesting that an upregulation of this gene could be relevant in MS pathogenesis. *TBKBP1* codes for the TBK1 binding protein 1, an adaptor protein able to bind TBK1 and involved in the TNFα/NFkB pathway [35]. Contrary to *TBKBP1*, for which few reports are available regarding its function*, TBK1* is involved in several immune processes, including T-cell homeostasis and migration cells from lymph nodes to the CNS in the MS murine model [36]. Although the direct involvement of TBKBP1 in TBK1-mediated signaling in migration of T cells has never been investigated, we could not exclude that an alteration of the expression of this gene could also influence this mechanism. Moreover, TBKBP1 was recently found to be an important regulator of development and survival of natural killer (NK) cells [37]. The role of NK cells in MS is still debated, however there is increasing evidence that certain subtypes of NK cells are able to modulate other immune cells and could be involved in autoimmune processes [38, 39]. Supporting these hypotheses, *TBKBP1* is highly expressed in activated CD4^+^ and CD8^+^ T cells, as well as NK cells (Online Resource 1 Fig. S6). Further tailored experiments are needed to elucidate the involvement of *TBKBP1* in MS.

Concluding, our results suggest that no novel signal associated with MS with a strong effect and specific for the continental Italian population exists. Nevertheless, we prioritized two loci, already identified to be associated with MS, that may have ethnic specificity in the Italian population, one of them pointing to the *TBKBP1* gene as a candidate to be investigated further.

## Supplementary Information

Below is the link to the electronic supplementary material.

Supplementary file1 Online Resource 1: supplementary methods and figures. Online Resource 2: association results according to the “genome-wide Italian approach.” Online Resource 3: association results according to the “meta-analysis approach.” Online Resource 4: association results of target sequencing.Online Resource 5: independent eQTL and mQTL associations in MS-related tissues (DOCX 11035 kb)

Supplementary file1 Online Resource 1: supplementary methods and figures. Online Resource 2: association results according to the “genome-wide Italian approach.” Online Resource 3: association results according to the “meta-analysis approach.” Online Resource 4: association results of target sequencing.Online Resource 5: independent eQTL and mQTL associations in MS-related tissues (DOCX 27 kb)

Supplementary file1 Online Resource 1: supplementary methods and figures. Online Resource 2: association results according to the “genome-wide Italian approach.” Online Resource 3: association results according to the “meta-analysis approach.” Online Resource 4: association results of target sequencing.Online Resource 5: independent eQTL and mQTL associations in MS-related tissues (DOCX 34 kb)

Supplementary file1 Online Resource 1: supplementary methods and figures. Online Resource 2: association results according to the “genome-wide Italian approach.” Online Resource 3: association results according to the “meta-analysis approach.” Online Resource 4: association results of target sequencing.Online Resource 5: independent eQTL and mQTL associations in MS-related tissues (DOCX 44 kb)

Supplementary file1 Online Resource 1: supplementary methods and figures. Online Resource 2: association results according to the “genome-wide Italian approach.” Online Resource 3: association results according to the “meta-analysis approach.” Online Resource 4: association results of target sequencing.Online Resource 5: independent eQTL and mQTL associations in MS-related tissues (DOCX 29 kb)
