# Correction to: A multi-step genomic approach prioritized TBKBP1 gene as relevant for multiple sclerosis susceptibility

**DOI:** 10.1007/s00415-022-11216-6

**Published:** 2022-06-25

**Authors:** Melissa Sorosina, Nadia Barizzone, Ferdinando Clarelli, Santosh Anand, Sara Lupoli, Erika Salvi, Eleonora Mangano, Roberta Bordoni, Tina Roostaei, Elisabetta Mascia, Miriam Zuccalà, Domizia Vecchio, Paola Cavalla, Silvia Santoro, Laura Ferrè, Alen Zollo, PROGEMUS, Lucia Florio, Paolo Ragonese, Alberto Gajofatto, Elio Scarpini, Domenico Caputo, Claudio Gasperini, Franco Granella, Paola Cavalla, Roberto Bergamaschi, Giovanni Ristori, Claudio Solaro, Filippo Martinelli Boneschi, Francesco Passantino, Maura Pugliatti, Antonio Gallo, Laura Brambilla, Marinella Clerico, Fioravante Capone, Maria Trojano, Cristina Barlassina, Daniele Cusi, Vittorio Martinelli, Giancarlo Comi, Maurizio Leone, Massimo Filippi, Nikolaos A. Patsopoulos, Philip L. De Jager, Gianluca De Bellis, Federica Esposito, Sandra D’Alfonso, Filippo Martinelli Boneschi

**Affiliations:** 1 Laboratory of Human Genetics of Neurological Disorders, Division of Neuroscience, IRCCS San Raffaele Scientific Institute, 20132 Milan, Italy; 2 Department of Health Sciences, Interdisciplinary Research Center of Autoimmune Diseases (IRCAD), University of Eastern Piedmont, Avogadro University, 28100 Novara, Italy; 3 Department of Informatics, Systems and Communications (DISCo), University of Milano-Bicocca, Milan, Italy; 4 Department of Health Sciences, University of Milan, 20139 Milan, Italy; 5 Neuroalgology Unit, Fondazione IRCCS Istituto Neurologico “Carlo Besta”, 20133 Milan, Italy; 6 National Research Council of Italy, Institute for Biomedical Technologies, Segrate, 20090 Milan, Italy; 7 Center for Translational and Computational Neuroimmunology, Department of Neurology and the Taub Institute for Research On Alzheimer’s Disease and the Aging Brain, Columbia University Irving Medical Center, New York, NY 10032 USA; 8 MS Centre, SCDU Neurology, AOU Maggiore Della Carità, Department of Translational Medicine, Interdisciplinary Research Center of Autoimmune Diseases (IRCAD), University of Eastern Piedmont Avogadro, 28100 Novara, Italy; 9 MS Center, Department of Neuroscience and Mental Health, City of Health and Science University Hospital of Torino, 10126 Turin, Italy; 10 Neurology Unit, IRCCS San Raffaele Scientific Institute, 20132 Milan, Italy; 11 Department of Pathophysiology and Transplantation (DEPT), Dino Ferrari Centre, Neuroscience Section, University of Milan, 20122 Milan, Italy; 12 Bio4Dreams, Business Nursery for Life Sciences, Piazzale Principessa Clotilde 4/A, 20121 Milan, Italy; 13 SC Neurologia, Dipartimento Di Scienze Mediche, IRCCS Casa Sollievo Della Sofferenza, San Giovanni Rotondo, Italy; 14 Vita-Salute San Raffaele University, 20132 Milan, Italy; 15 Neuroimaging Research Unit, Division of Neuroscience, IRCCS San Raffaele Scientific Institute, 20132 Milan, Italy; 16 Neurophysiology Unit, IRCCS San Raffaele Scientific Institute, IRCCS San Raffaele Scientific Institute, 20132 Milan, Italy; 17 Systems Biology and Computer Science Program, Ann Romney Center for Neurological Diseases, Department of Neurology, Brigham and Women’s Hospital, Boston, MA 02115 USA; 18 Division of Genetics, Department of Medicine, Harvard Medical School, Brigham and Women’s Hospital, Boston, MA 02115 USA; 19 Harvard Medical School, Boston, MA 02115 USA; 20 Broad Institute of Harvard and Massachusetts Institute of Technology, Cambridge, MA USA; 21 Neurology Unit and MS Centre, Foundation IRCCS Ca’ Granda Ospedale Maggiore Policlinico, Via Francesco Sforza 35, 20122 Milan, Italy

## Correction to: Journal of Neurology 10.1007/s00415-022-11109-8

The original version of this article unfortunately contained a mistake. The table 2 caption should read as

**Table 2 Association results in the replication sample**. For each SNP, the association data in the replication cohort and results from the meta-analyses between discovery and replication cohorts (including and not including the NonIT-EUR cohort) are reported. Results for the genome-wide Italian approach are reported in the upper panel (A), while results for the approaches including the NonIT-EUR are reported in panel B (discovery sample derived from ITA_GWAS_ alone) and C (SNPs identified through the meta-analysis between ITA_GWAS_ and ITA_iChip_). A1: reference allele (the same as defined in table 1); OR: odds ratio; L95: lower-bound of 95%-confidence interval; U95: upper bound of 95%-confidence interval; P: P-value of association; I^2^: heterogeneity index. The SNPs with an association beyond the multiple testing correction (Bonferroni threshold of P-value: 1.7 × 10^–3^, 3.6 × 10^–3^, 6.25 × 10^–3^ respectively for A, B and C) are highlighted in bold. No significant association was found in the replication cohort for SNPs selected through the Italian genome-wide approach.

The original article has been corrected.

