# Supplementary material for: A multi-step genomic approach prioritized TBKBP1 gene as relevant for multiple sclerosis susceptibility
Source: J Neurol. 2022 May 12;269(8):4510–22. doi: 10.1007/s00415-022-11109-8 (PMC9294010; doi:10.1007/s00415-022-11109-8)
Supplement: Supplementary file 1 — Supplementary file1 Online Resource 1: supplementary methods and figures. Online Resource 2: association results according to the “genome-wide Italian approach.” Online Resource 3: association results according to the “meta-analysis approach.” Online Resource 4: association results of target sequencing.Online Resource 5: independent eQTL and mQTL associations in MS-related tissues (DOCX 11035 kb) [file 415_2022_11109_MOESM1_ESM.docx]

**Supplementary information**

**A multi-step genomic approach prioritized TBKBP1 gene as relevant for MS susceptibility**

Melissa Sorosina^1^*, Nadia Barizzone^2^*, Ferdinando Clarelli^1^, Santosh Anand^3^, Sara Lupoli^4^, Erika Salvi^4,5^, Eleonora Mangano^6^, Roberta Bordoni^6^, Tina Roostaei^7^, Elisabetta Mascia^1^, Miriam Zuccalà^2^, Domizia Vecchio^8^, Paola Cavalla^9^, Silvia Santoro^1^, Laura Ferrè^1,10^, Mattia Pozzato^11,12^, PROGEMUS, Cristina Barlassina^4^, Daniele Cusi^6,13^, Vittorio Martinelli^10^, Giancarlo Comi^10^, Maurizio Leone^14^, Massimo Filippi^10,15-17^, Nikolaos A. Patsopoulos^18-21^, Philip L. De Jager^7^, Gianluca De Bellis^6^, Federica Esposito^1,10^§, Sandra D'Alfonso^2^§, Filippo Martinelli Boneschi^11,12^§

^1^ Laboratory of Human Genetics of Neurological Disorders, Institute of Experimental Neurology, IRCCS San Raffaele Scientific Institute, 20132, Milan, Italy

^2^ Department of Health Sciences, Interdisciplinary Research Center of Autoimmune Diseases (IRCAD), University of Eastern Piedmont, Avogadro University, 28100, Novara, Italy

^3^ Department of Informatics, Systems and Communications (DISCo), University of Milano-Bicocca, Milan, Italy

^4^ Department of Health Sciences, University of Milan, 20139, Milan, Italy.

^5^ Neuroalgology Unit, Fondazione IRCCS Istituto Neurologico “Carlo Besta”, 20133, Milan, Italy.

^6^ National Research Council of Italy, Institute for Biomedical Technologies, 20090, Segrate, Milan, Italy

^7^ Center for Translational &Computational Neuroimmunology, Department of Neurology and the Taub Institute for Research on Alzheimer’s Disease and the Aging Brain, Columbia University Irving Medical Center, New York, NY, 10032, USA

^8^ MS Centre, SCDU Neurology, AOU Maggiore della Carità, Department of Translational Medicine, Interdisciplinary Research Center of Autoimmune Diseases (IRCAD), University of Eastern Piedmont Avogadro, 28100, Novara, Italy

^9^ MS Center, Department of Neuroscience and Mental Health, City of Health and Science University Hospital of Torino, Via Cherasco 15, 10126, Turin, Italy

^10^ Neurology Unit, IRCCS San Raffaele Scientific Institute, Via Olgettina 48, 20132, Milan, Italy.

^11^ Department of Pathophysiology and Transplantation (DEPT), Dino Ferrari Centre, Neuroscience Section, University of Milan, Via Francesco Sforza 35, 20122, Milan, Italy

^12^ Neurology Unit and MS Centre, Foundation IRCCS Ca' Granda Ospedale Maggiore Policlinico, Via Francesco Sforza 35, 20122, Milan, Italy

^13^ Bio4Dreams - Business Nursery for Life Sciences, Piazzale Principessa Clotilde 4/A, 20121, Milan, Italy

^14^ SC Neurologia, Dipartimento di Scienze Mediche, IRCCS Casa Sollievo della Sofferenza, San Giovanni Rotondo, Italy

^15^ Vita-Salute San Raffaele University, Via Olgettina 48, 20132, Milan, Italy

^16^ Neuroimaging Research Unit, Division of Neuroscience, IRCCS San Raffaele Scientific Institute, Via Olgettina 48, 20132, Milan, Italy

^17^ Neurophysiology Unit, IRCCS San Raffaele Scientific Institute, IRCCS San Raffaele Scientific Institute, Via Olgettina 48, 20132, Milan, Italy

^18^ Systems Biology and Computer Science Program, Ann Romney Center for Neurological Diseases, Department of Neurology, Brigham & Women's Hospital, Boston, 02115 MA, USA

^19^ Division of Genetics, Department of Medicine, Brigham & Women's Hospital, Harvard Medical School, Boston, MA, USA

^20^ Harvard Medical School, Boston, MA 02115, USA

^21^ Broad Institute of Harvard and Massachusetts Institute of Technology, Cambridge, MA, USA

* equal contribution

§ equal contribution

° Membership of the consortia are listed in the co-investigator appendix

**Corresponding author:**

Filippo Martinelli Boneschi

Neurology Unit and MS Centre, Foundation IRCCS Ca' Granda Ospedale Maggiore Policlinico, Via Francesco Sforza 35, 20122 Milan, Italy

**Table of content**

*Supplementary Methods*……………………………………………………………………………… page 2

*Supplementary Figures*………………………………………………………………………………. page 6

*Supplementary References* ……………………………………………………………………………page 12

**Supplementary Methods**

*Discovery: ITA_GWAS_ case-control sample*

MS samples belonging to the ITA_GWAS_ cohort were genotyped at genome-wide level at the Wellcome Trust Sanger Institute using the Illumina® Human660-Quad chip[1]. Quality controls (QC) at SNP level included: SNPs call rate >0.99 and a MAF (Minor Allele Frequency) >0.01. Quality controls at individual level included: gender mismatch, presence of duplicates and cryptic relatedness, call rate <95%, and heterozygosity <±3SD (standard deviation) from mean level. SNPs and samples failing the QC step, as well as SNPs on sexual chromosomes, were removed from the analyses. Healthy individuals belonging to the ITA_GWAS_ cohort were genotyped at the University of Milan, using the Illumina® Infinium 1M-duo BeadChips. All quality controls were performed in accordance with the protocol written by Anderson et al[2].

Population stratification was assessed with principal component analysis (PCA), using SNPs in common between the two arrays (547,937 SNPs), as implemented in the EIGENSOFT package (version 3.0)[3]. We excluded outlier samples defined as individuals exceeding a default number of 6 SDs along one of the top 10 PCs, from the whole sample. We selected the first 10 PCs to include them as covariates in the logistic regression model and ruled out relatedness across subjects through identity-by-descent analysis, as implemented in PLINK[4], for all possible pairs of individuals. For both MS and HC samples, we used markers of highest quality (SNPs call rate >0.99, MAF >0.01) to impute approximately 30 million SNPs, against 1000 Genomes Phase I haplotypes ALL reference panel (release March 2012), using Minimac software[5].

The two full imputed datasets were then merged, and the quality of imputation was estimated from dosage data using r2_hat software (http://csg.sph.umich.edu/yli/software.html). Imputed SNPs with a MAF <0.01 and with poor or modest imputation quality (R^2^ <0.80) were removed before association analysis. We then computed each SNP p-value with a logistic regression of MS status on SNP dosage levels, adjusting for sex and the first 10 principal components. Imputation and single-marker significance of association was estimated using mach2dat software[6].

*Discovery: ITA_iChip_ case-control sample*

Samples belonging to the ITA_iChip_ cohort were genotyped at the Wellcome Trust Sanger Institute using the Illumina® Immunochip covering 196,524 SNPs and 184 non-HLA genomic regions with potential immunological function[7]. Quality controls at SNP and individual levels were performed as previously described[7]. Single-marker allelic association analyses was performed in PLINK v1.07, fitting logistic regression model including gender as covariate.

*Discovery: NonIT-EUR case-control sample*

The “NonIT-EUR” collection of subjects is a combination of 6 individual sample collections, for a total of 4,088 MS patients and 7,144 HC of European descent, previously described[8]. Genotyping of each data set belonging to the NonIT-EUR cohort was performed using different genotyping platforms, as previously described[8]. Data are available at the database of Genotypes and Phenotypes (dbGaP) website under the following codes: phs000275.v1.p1, phs000294.v1.p1, phs000171.v1.p1, phs000139.v1.p1. The ANZGENE GWAS data is available via request to the ANZGENE Consortium. A direct request can be made via MSRA.org.au. Each data set was processed as described here[9]. In brief, SNPs belonging to the sex chromosomes, with a missingness rate ≥0.01, a differential missingness between cases and controls with p-value <0.001, MAF <1%, which violated the Hardy–Weinberg equilibrium (HWE) in controls (different MAF-stratified p-value cut-offs were used: for MAF >0.3: p-value <0.0001; for MAF between 0.2 and 0.3: p-value <0.00001; for MAF between 0.1 and 0.2: p-value <0.000001; for MAF <0.1: p-value <0.000001) were removed. Individuals with missingness rate ≥0.05 were removed, as well as individuals with inbreeding coefficient of F ≥0.05 or F ≤-0.05 (calculated on LD-pruned SNPs), related to 3^rd^-4^th^ degree. EIGENSOFT[10] was used to calculate PCs and individuals that were outside ±6 SD on each of the 10 first PCs were removed. Moreover, samples were projected onto the HapMap 3 populations and individuals that fell outside the main cluster of the respective data set or the main European-ancestry cluster were manually removed. BEAGLE v3.2.1[11] and the 1000 Genomes Phase Ia European panel were used to impute ~34 million genetic variants, as previously described[9].

The post-imputation probabilities were used to perform logistic regression within each of the data sets, using the first 5 PCs as covariates to account for population stratification. Monomorphic SNPs as well as SNPs with MAF <1% or low imputation quality (INFO score <0.1) were removed. Summary statistics were then combined using a fixed effects meta-analysis[9].

*Discovery: statistical tests*

For the “genome-wide Italian approach”, single-SNP logistic regression using sex as covariate was conducted under additive model of inheritance.

For the “meta-analysis approach”, inverse-variance weighting meta-analysis between cohorts was performed using PLINK[4] under a fixed-effect model. NonIT-EUR cohort was treated as individual study when meta-analyzed with the Italian cohorts.

*Replication of associated signals*

Starting from the signals of association identified through the discovery step (p<5x10^-7^), we constructed loci using a ±100 kb window centred on the top signal and an r^2^ of 0.4 according to CEU data from the 1000 Genomes Project (www.1000genomes.org). For each locus the most associated SNP and, if present, a secondary associated SNP (P<5x10^-7^), prioritizing the ones with the lower linkage disequilibrium with the top SNP, were selected and tested for association in the Italian cohort ITA_OA_. Overall, a total of 60 SNPs was chosen for the replication step (Table S1 and S2).

Genotyping was performed using custom and pre-designed TaqMan assays (Thermo Fisher Scientific, Waltham, USA) assembled on two OpenArray chips run on the TaqMan Open Array Genotyping System (Thermo Fisher Scientific, Waltham, USA) or with the Illumina MS replication chip (Illumina, San Diego, USA), a custom array covering more than 300,000 SNPs in MS associated loci[9].

After failing of the design of few TaqMan assays, the following substitutions were performed: the top associated SNP of locus 6 (rs2744155) was replaced by its best proxy rs2729590 (r^2^=1); rs11859698 (locus 7) was replaced by rs741175, rs7224600 (locus 26) was replaced by rs35493137; rs2353064, the only signal of region 4, failed the design of the assay and it was not replaced, as well as rs427221 (locus 1), rs138458584 (locus 24), rs11084505 (locus 29) and rs2426300 (locus 30).

Genotyping on the Open Array Genotyping System was performed according to the manufacturer’s instructions; the autocalling method as implemented in the TaqMan Genotyper software version 1.3 was used to assign genotypes. Samples with call rate <80% and SNPs with call rate <90% and failing HWE test at p<0.0001 were removed from the association analysis.

Single-SNP logistic regression using gender as covariate was conducted under additive model of inheritance, using PLINK[4]. Given the partial overlap between the ITA_iChip_ and ITA_OA_ cohorts (figure S1), overlapping individuals were removed from the analyses regarding SNPs derived from the analysis including the ITA_iChip_ cohort.

Meta-analyses between the discovery and replication cohorts were performed using PLINK[4] assuming a fixed-effect model.

*Next-generation sequencing*

Pooled-sequencing (84 pools, 12 individuals per pool) was performed as described elsewhere[12] using the Agilent SureSelect target enrichment method (Agilent Technologies, Santa Clara, USA) according to the manufacturer’s protocol. LD block containing the replicated associated signals (locus 1 and locus 8) was defined according to HapMap rel 22 (CEU (Utah residents with ancestry from northern and western Europe) only)[13] using Haploview software[14] and sequenced. Briefly, DNA quantity has been properly balanced in each pool in order to equally represent each genome; after the fragmentation of DNA in each pool using the Covaris shearing system (Covaris inc., Massachusetts, USA), ligation to specific paired-end adaptors, preparation of amplified libraries and their hybridization to capture probes were performed. Libraries were then sequenced through paired-end multiplexed sequencing on the Illumina GaIIx platform (Illumina, San Diego, USA), combining 6 pools tagged with different index sequences in each lane and producing 2 x 85 bp read lengths[12].

The bioinformatics pipeline and variant calling were performed as previously described[12]. Briefly, CRISP caller[15] was used to call the variants and a quality filter was applied to remove false positives. The cumulative allele frequencies (AF) in patients and controls have been estimated using an ad-hoc custom pipeline, which was developed to guarantee accurate AF estimation from pooled-sequencing, by filtering out spurious variants resulting from sequencing noise[12]. Specifically, an empirically determined threshold (2.6%) was applied to single pool alternative AF to remove spurious reads[12].

Only variants with a quality score >100 were included in the analyses. Fisher exact test comparing MS and HC was performed on allelic counts.

The regional association plots were generated using LocusZoom (http://locuszoom.sph.umich.edu)[16].

*Conditional cis eQTL analyses*

Expression and genotyping data were obtained from GTEx Portal (www.gtexportal.org)[17] (release phs000424.v6.p1): post processed imputed genotypes (individual call rate >95%, variant info score threshold>0.4, and MAF≥1%) as well as processed, filtered and normalized gene expression matrices of up to 450 individuals and 7 tissues (whole blood, Brain Frontal Cortex (BA9), Brain Cortex, Cerebellar Hemisphere, Cerebellum, Anterior cingulate cortex (BA24), Hypothalamus; number of samples varies based on the analysed tissue) were downloaded and used for the analyses. Additionally, a set of covariates which include the top 3 genotyping principal components, a set of PEER (Probabilistic Estimation of Expression Residuals) factors explaining expression variability identified using the PEER method[18], the genotyping platform and sex were already available within the GTEx project and were included as covariates in the analyses. Conditional *cis* eQTL was performed using QTLtools v1.0[19] testing the association of each variant-gene pairing according to a window of ±1Mb from the transcription start site and accounting for the mentioned covariates. An adaptive permutation approach (1000 to 10000 permutations) was used followed by a forward-backward stepwise regression step to identify the number of independent signals and the best candidate.

*Conditional cis mQTL analyses*

Samples from 156 MS patients from the Comprehensive Longitudinal Investigation of Multiple Sclerosis at the Brigham and Women's Hospital (CLIMB) study[20] were used for the mQTL analysis. Patients were of European ancestry, they had a relapsing-remitting disease course, they were in remission and treated with either glatiramer acetate or dimethyl fumarate at the time of blood sampling. Frozen PBMC from participants were used to purify CD4^+^ T cells. Extracted DNA was used for DNA methylation measurements using the Infinium MethylationEPIC array (Illumina, San Diego, USA). Quality control was performed using R minfi[21] package. Probes with beadcount <3 in >5% of samples or detection p-value >0.01 in any sample, in addition to methylation sites located on sex chromosomes, sites associated with probes with polymorphic targets with minor allele frequency >1% in individuals with European ancestry, and sites associated with cross-reactive probes were excluded from the study[22]. Normalization was performed using Noob[23] and BMIQ[24] algorithms. Methylation M-values were calculated and quantile-normalized prior to statistical analysis. Genotyping was performed using either Illumina MEGA-EX (Illumina, San Diego, USA) or Affymetrix 6.0 arrays (Affymetrix, Santa Clara, USA). Quality control was performed using PLINK[4]. SNPs with call rate <95% and samples with genotyping rate <90%, mismatch between recorded sex and genetic sex, low or high heterozygosity rates (>3 SD) and increased relatedness (pi-hat >0.125) were removed. Imputation was performed using the Michigan Imputation Server[25] and the Haplotype Reference Consortium (HRC) panel v1.1. SNPs with low imputation quality (R^2^ <0.8), minor allele frequency <0.05, minor allele count <15, and departure from Hardy-Weinberg equilibrium (p <1×10^-6^) were excluded from analysis. *Cis* mQTL analysis was performed genome-wide using QTLtools[19] Briefly, linear regression models were fit between each DNA methylation site and all SNPs in cis (±1Mb), accounting for the effects of age, sex, treatment, genotyping array, the first 3 genotyping principle components (PCs) and the first 4 methylation PCs. Nominal p-values for the top variants in cis were adjusted for the number of tests performed in cis using a permutation scheme (n=1000 permutations). To account for multiple CpG sites tested across the whole genome, mQTL effects were considered significant at 5% False Discovery Rate (FDR). Conditional analysis was performed for FDR-significant CpGs in order to identify additional independent cis-mQTL effects other than the effects of the top associated variant.

*Chromatin state annotation*

Publicly available datasets of chromatin state annotations were used to predict the function of the genomic regions of interest. Specifically, the 25-state model based on combinations of histone modification marks across 127 reference epigenomes obtained within the Roadmap Epigenomics[26] and ENCODE projects[27] was evaluated for MS-related tissues (immune tissues, CNS tissues) and visualized through the WashU Epigenome Browser ([http://epigenomegateway.wustl.edu](http://epigenomegateway.wustl.edu/))[28].

*Evaluation of TBKBP1 expression across immune cell types*

The expression of *TBKBP1* across immune cells was evaluated taking advantage of the DICE dataset (Database of Immune Cell Expression, Expression quantitative trait loci [eQTLs], and Epigenomics) (https://dice-database.org)[29], a repository of transcription data of 15 immune human cell types: naive B cells, naive CD4^+^ T cells, naive CD8^+^ T cells, and naive regulatory T cells (TREG), six CD4^+^ memory or more differentiated T cell subsets (TH1, TH1/17, TH17, TH2, follicular helper T cell (TFH), and memory TREG), two activated cell types (naive CD4^+^ and CD8^+^ T cells that were stimulated *ex vivo*). Transcript per millions (TPM) values for TBKBP1 for each cell type, as well as differential expression results (adjusted P values, Fold changes) calculated by the DESeq package (version 1.6.3) of pairwise comparison between cell types were downloaded and plotted.

**Supplementary Figures**

**
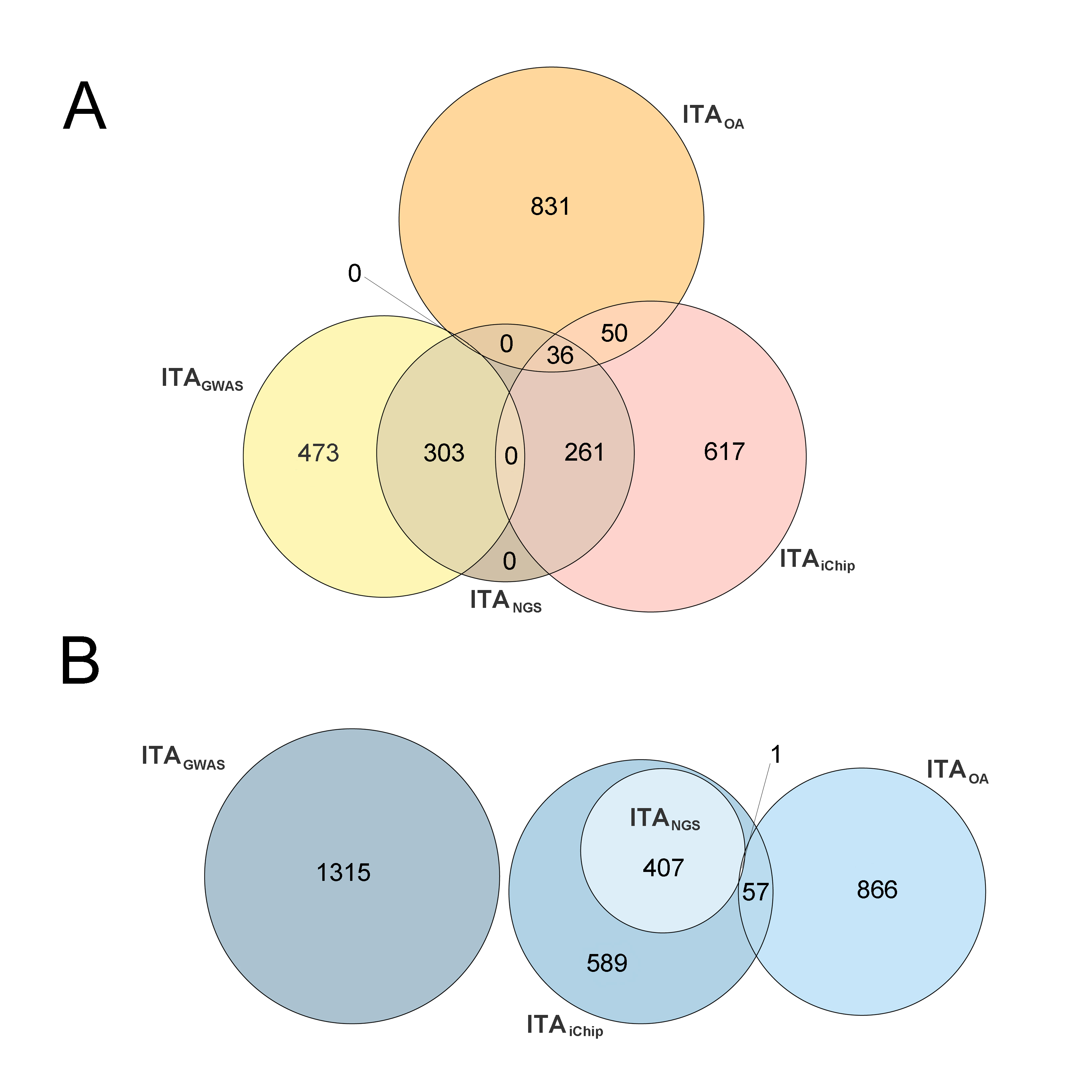
**

**Figure S1. Overlap among Italian cohorts.** Overlap among individuals belonging to the Italian cohorts is shown for A) MS patients and B) healthy individuals. Dimension of the circles is proportional to the sample size. Euler diagrams were generated through the *eulerr* package in R environment[30].

**Figure S2. Genome-wide association analysis in the ITA_GWAS_ cohort**. Association results of the GWAS in the ITA_GWAS_ cohort are plotted as negative log-transformed p-values against their position in the genome. Odd chromosomes are in grey, while even chromosomes in blue. Red line indicates the 5x10^-8^ p-value threshold, while the orange line indicates the 5x10^-6^ p-value threshold. Chr: chromosome

**
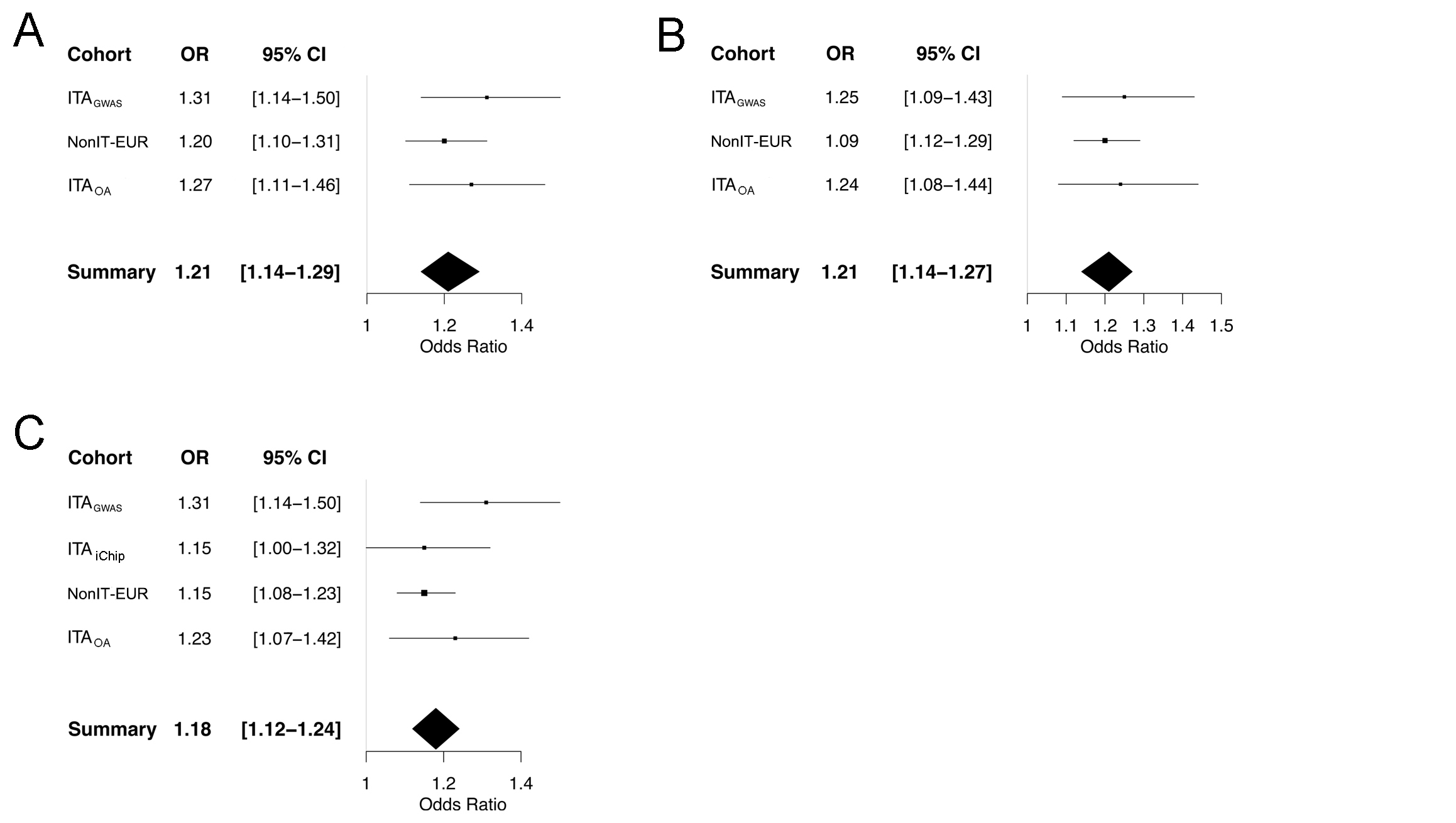
**

**Figure S3. Meta-analysis across the discovery and replication datasets**. The forest plots summarize the results obtained for rs338603 (A), rs8070463 (B) and rs669607 (C) in the ITA_GWAS_, ITA_iChip_, NonIT-EUR and ITA_OA_ replication cohorts. Summary ORs and 95% Confidence Intervals (CI) were calculated using the fixed-effect method.


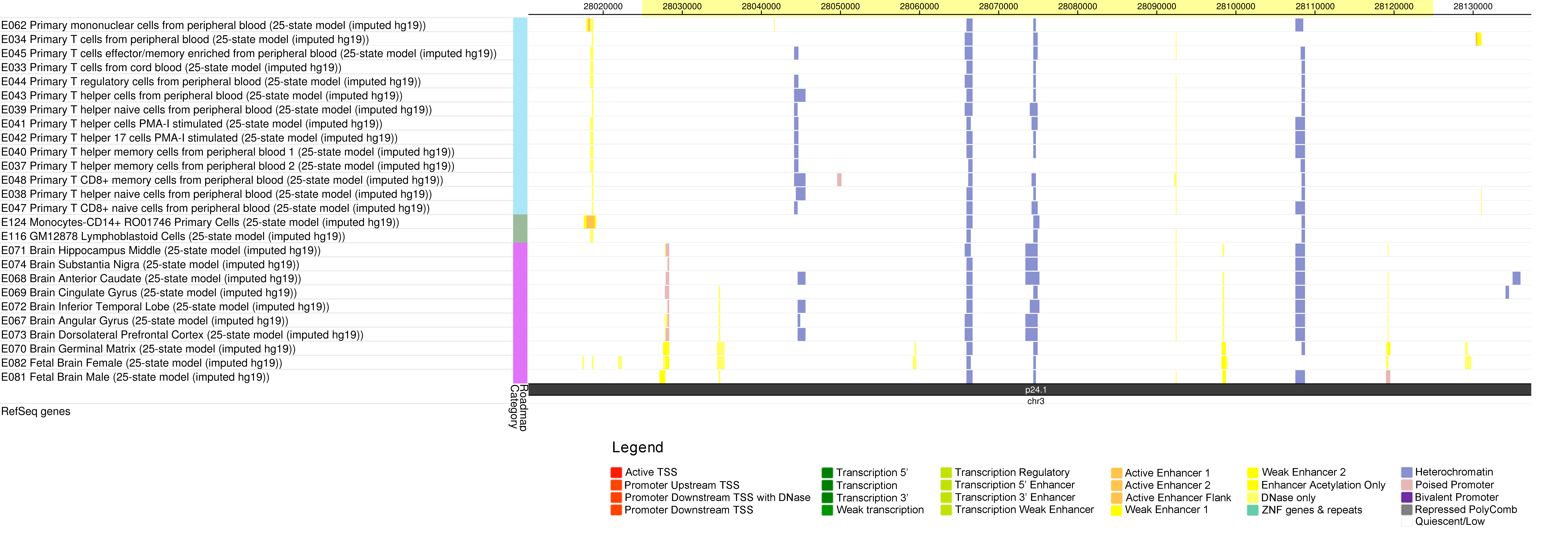


**Figure S4. Chromatin state prediction of locus 1 on chromosome 3**. Marks of chromatin segmentation defined by Roadmap Epigenomics for 26 cell types are reported for locus 1 (highlighted in yellow). Specifically, 14 immune-related cell types (light blue according to “roadmap category” column), 2 immune-related cell types from Roadmap-processed ENCODE data (green according to “roadmap category” column) and 10 brain-related cell types (purple according to “roadmap category” column) were analysed. Each colour codes for a different genomic function according to the legend below. No RefSeq genes are present in the locus. Genomic position is defined according to hg19.

**
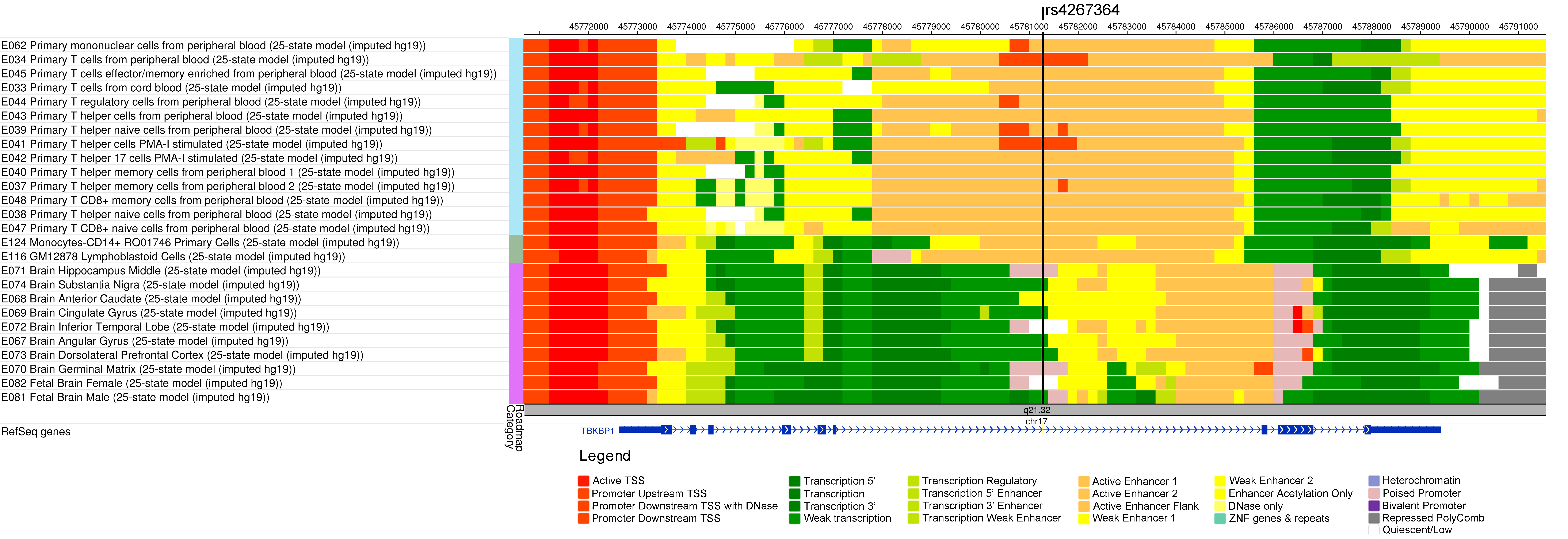
**

**Figure S5. Chromatin state prediction of genomic region of rs4267364 in locus 8 on chromosome 17**. The genomic rs4267364 locus is shown according to marks of chromatin segmentation defined by Roadmap Epigenomics for 26 cell types. Specifically, 14 immune-related cell types (light blue according to “roadmap category” column), 2 immune-related cell types from Roadmap-processed ENCODE data (green according to “roadmap category” column) and 10 brain-related cell types (purple according to “roadmap category” column) were analysed. Each colour codes for a different genomic function according to the legend below. RefSeq genes of the region is reported in the lower track. The black vertical line indicates the position of rs4267364. Genomic position is defined according to hg19.

A

B

**Figure S6. Expression of TBKBP1 across immune cells**. A) TBKBP1 expression levels across 15 immune cell types according to the DICE database. Levels are expressed as transcripts per million (TPM) and represented as box-plots (Tukey method). Each dot represents one sample. B) Differential gene expression across cell types as calculated within the DICE database. The matrix indicates results from pair-wise comparisons of two cell types on the y- (cell type 1) and x-axis (cell type 2), with log2 Fold change coloured according to the legend. For each comparison, the p-value is shown.

**Supplementary References**

1. International Multiple Sclerosis Genetics Consortium, Wellcome Trust Case Control Consortium 2, Sawcer S, et al (2011) Genetic risk and a primary role for cell-mediated immune mechanisms in multiple sclerosis. Nature 476:214–219

2. Anderson CA, Pettersson FH, Clarke GM, et al (2010) Data quality control in genetic case-control association studies. Nat Protoc 5:1564–1573. https://doi.org/10.1038/nprot.2010.116

3. Patterson N, Price AL, Reich D (2006) Population structure and eigenanalysis. PLoS Genet 2:2074–2093. https://doi.org/10.1371/journal.pgen.0020190

4. Purcell S, Neale B, Todd-Brown K, et al (2007) PLINK: a tool set for whole-genome association and population-based linkage analyses. Am J Hum Genet 81:559–75. https://doi.org/10.1086/519795

5. Howie B, Fuchsberger C, Stephens M, et al (2012) Fast and accurate genotype imputation in genome-wide association studies through pre-phasing. Nat Genet 44:955–9. https://doi.org/10.1038/ng.2354

6. Li Y, Willer CJ, Ding J, et al (2010) MaCH: using sequence and genotype data to estimate haplotypes and unobserved genotypes. Genet Epidemiol 34:816–34. https://doi.org/10.1002/gepi.20533

7. International Multiple Sclerosis Genetics Consortium (IMSGC), Beecham AH, Patsopoulos NA, et al (2013) Analysis of immune-related loci identifies 48 new susceptibility variants for multiple sclerosis. Nat Genet 45:1353–60. https://doi.org/10.1038/ng.2770

8. Patsopoulos NA, Bayer Pharma MS Genetics Working Group, Steering Committees of Studies Evaluating IFNβ-1b and a CCR1-Antagonist, et al (2011) Genome-wide meta-analysis identifies novel multiple sclerosis susceptibility loci. Ann Neurol 70:897–912. https://doi.org/10.1002/ana.22609

9. International Multiple Sclerosis Genetics Consortium (IMSGC) (2019) Multiple sclerosis genomic map implicates peripheral immune cells and microglia in susceptibility. Science (80- ) 365:eaav7188. https://doi.org/10.1126/science.aav7188

10. Price AL, Patterson NJ, Plenge RM, et al (2006) Principal components analysis corrects for stratification in genome-wide association studies. Nat Genet 38:904–9. https://doi.org/10.1038/ng1847

11. Browning SR, Browning BL (2011) Population structure can inflate SNP-based heritability estimates. Am J Hum Genet 89:191–3; author reply 193-5. https://doi.org/10.1016/j.ajhg.2011.05.025

12. Anand S, Mangano E, Barizzone N, et al (2016) Next Generation Sequencing of Pooled Samples: Guideline for Variants’ Filtering. Sci Rep 6:33735. https://doi.org/10.1038/srep33735

13. International HapMap Consortium, Frazer KA, Ballinger DG, et al (2007) A second generation human haplotype map of over 3.1 million SNPs. Nature 449:851–61. https://doi.org/10.1038/nature06258

14. Barrett JC, Fry B, Maller J, Daly MJ (2005) Haploview: Analysis and visualization of LD and haplotype maps. Bioinformatics 21:263–265. https://doi.org/10.1093/bioinformatics/bth457

15. Bansal V (2010) A statistical method for the detection of variants from next-generation resequencing of DNA pools. Bioinformatics 26:i318-24. https://doi.org/10.1093/bioinformatics/btq214

16. Pruim RJ, Welch RP, Sanna S, et al (2011) LocusZoom: Regional visualization of genome-wide association scan results. In: Bioinformatics. pp 2336–2337

17. GTEx Consortium (2013) The Genotype-Tissue Expression (GTEx) project. Nat Genet 45:580–5. https://doi.org/10.1038/ng.2653

18. Stegle O, Parts L, Durbin R, Winn J (2010) A bayesian framework to account for complex non-genetic factors in gene expression levels greatly increases power in eQTL studies. PLoS Comput Biol 6:1–11. https://doi.org/10.1371/journal.pcbi.1000770

19. Delaneau O, Ongen H, Brown AA, et al (2017) A complete tool set for molecular QTL discovery and analysis. Nat Commun 8:15452. https://doi.org/10.1038/ncomms15452

20. Gauthier SA, Glanz BI, Mandel M, Weiner HL (2006) A model for the comprehensive investigation of a chronic autoimmune disease: The multiple sclerosis CLIMB study. Autoimmun. Rev. 5:532–536

21. Aryee MJ, Jaffe AE, Corrada-Bravo H, et al (2014) Minfi: a flexible and comprehensive Bioconductor package for the analysis of Infinium DNA methylation microarrays. Bioinformatics 30:1363–9. https://doi.org/10.1093/bioinformatics/btu049

22. McCartney DL, Walker RM, Morris SW, et al (2016) Identification of polymorphic and off-target probe binding sites on the Illumina Infinium MethylationEPIC BeadChip. Genomics Data 9:22–24. https://doi.org/10.1016/j.gdata.2016.05.012

23. Triche TJ, Weisenberger DJ, Van Den Berg D, et al (2013) Low-level processing of Illumina Infinium DNA Methylation BeadArrays. Nucleic Acids Res 41:e90. https://doi.org/10.1093/nar/gkt090

24. Teschendorff AE, Marabita F, Lechner M, et al (2013) A beta-mixture quantile normalization method for correcting probe design bias in Illumina Infinium 450 k DNA methylation data. Bioinformatics 29:189–96. https://doi.org/10.1093/bioinformatics/bts680

25. Das S, Forer L, Schönherr S, et al (2016) Next-generation genotype imputation service and methods. Nat Genet 48:1284–1287. https://doi.org/10.1038/ng.3656

26. Roadmap Epigenomics Consortium, Kundaje A, Meuleman W, et al (2015) Integrative analysis of 111 reference human epigenomes. Nature 518:317–329. https://doi.org/10.1038/nature14248

27. ENCODE Project Consortium (2012) An integrated encyclopedia of DNA elements in the human genome. Nature 489:57–74. https://doi.org/10.1038/nature11247

28. Zhou X, Wang T (2012) Using the Wash U Epigenome Browser to examine genome-wide sequencing data. Curr Protoc Bioinforma Chapter 10:Unit10.10. https://doi.org/10.1002/0471250953.bi1010s40

29. Schmiedel BJ, Singh D, Madrigal A, et al (2018) Impact of Genetic Polymorphisms on Human Immune Cell Gene Expression. Cell 175:1701-1715.e16. https://doi.org/10.1016/j.cell.2018.10.022

30. Larson J, Jonathan A, Godfry R, et al (2018) Area-Proportional Euler and Venn Diagrams with Circles or Ellipses. R Packag version 410
